# Claudin-5 and inflammatory biomarkers in panic disorder: a 6-week follow-up study

**DOI:** 10.3389/fpsyt.2025.1703509

**Published:** 2025-12-11

**Authors:** Dilek Örüm, Zekiye Çatak, Ali Baran Tanrıkulu, Murad Atmaca

**Affiliations:** 1 Elazig Fethi Sekin City Hospital, Psychiatry, Elazig, Türkiye; 2 Elazig Fethi Sekin City Hospital, Medical Biochemistry, Elazig, Türkiye; 3 Elazig Mental Health and Diseases Hospital, Psychiatry, Elazig, Türkiye; 4 Fırat University, Psychiatry, Elazig, Türkiye

**Keywords:** panic disorder, Claudin-5, tight junction, blood-brain barrier, neuroinflammation

## Abstract

**Background:**

Claudin-5 is an important cell adhesion molecule of tight junctions in brain endothelial cells and plays an important role in the permeability of the blood–brain barrier (BBB). Panic disorder (PD) is a disorder characterized by increased neuroinflammatory processes that may result in increased BBB permeability. This study aimed to examine claudin-5 levels in PD at baseline and after a 6-week follow-up and to compare them with those of the healthy control (HC) group.

**Methods:**

Twenty-seven PD subjects (17 women and 10 men) and 25 HC subjects (15 women and 10 men) were included in this prospective cohort study.

**Results:**

Pre-treatment claudin-5 (p = 0.021), C-reactive protein (CRP) (p < 0.001), CRP/albumin ratio (p < 0.001), and neutrophil count (p < 0.001) were higher in the PD group than in the HC group. Claudin-5 levels (p = 0.001) and Panic Disorder Scale (PDS) scores (p < 0.001) of the PD group decreased significantly after 6 weeks of follow-up compared to those at baseline. Post-treatment claudin-5 levels of the PD group were similar to those of the HC group (p = 0.230). In the PD group, partial correlation analysis was performed by controlling for the effects of age, gender, body mass index (BMI), smoking status, antidepressant and benzodiazepine status, and a significant relationship was found between the pre-treatment PDS score and pre-treatment claudin-5 level (r = 0.474, p = 0.030). After various modeling attempts, a hierarchical model (controlling for age, gender, and BMI) was created using pre-treatment claudin-5 level and the aggregate index of systemic inflammation (sensitivity = 70.4%, specificity = 76.0%; Nagelkerke R^2^ = 0.468). The area under the receiver operating characteristic (ROC) curve of pre-treatment claudin-5 level for PD was 0.687 (p = 0.021).

**Conclusion:**

In subjects presenting with PD symptoms, increased parameters that may be associated with increased inflammation (such as CRP, CRP/albumin ratio, and neutrophil count), along with claudin-5 levels and the association between them, and the decrease in claudin-5 levels after a 6-week follow-up following a decrease in PD symptom severity, suggest that alterations in claudin-5 level in PD may be related to PD symptomatology. The high level of claudin-5 at the initial clinical presentation may be explained as a direct consequence of neuroinflammation in PD and/or as a compensatory change that occurs secondary to the neuroinflammation in PD. It can be assumed that as PD symptom severity decreases, neuroinflammation decreases, and claudin-5 production slows down.

## Introduction

**Figure 1:**
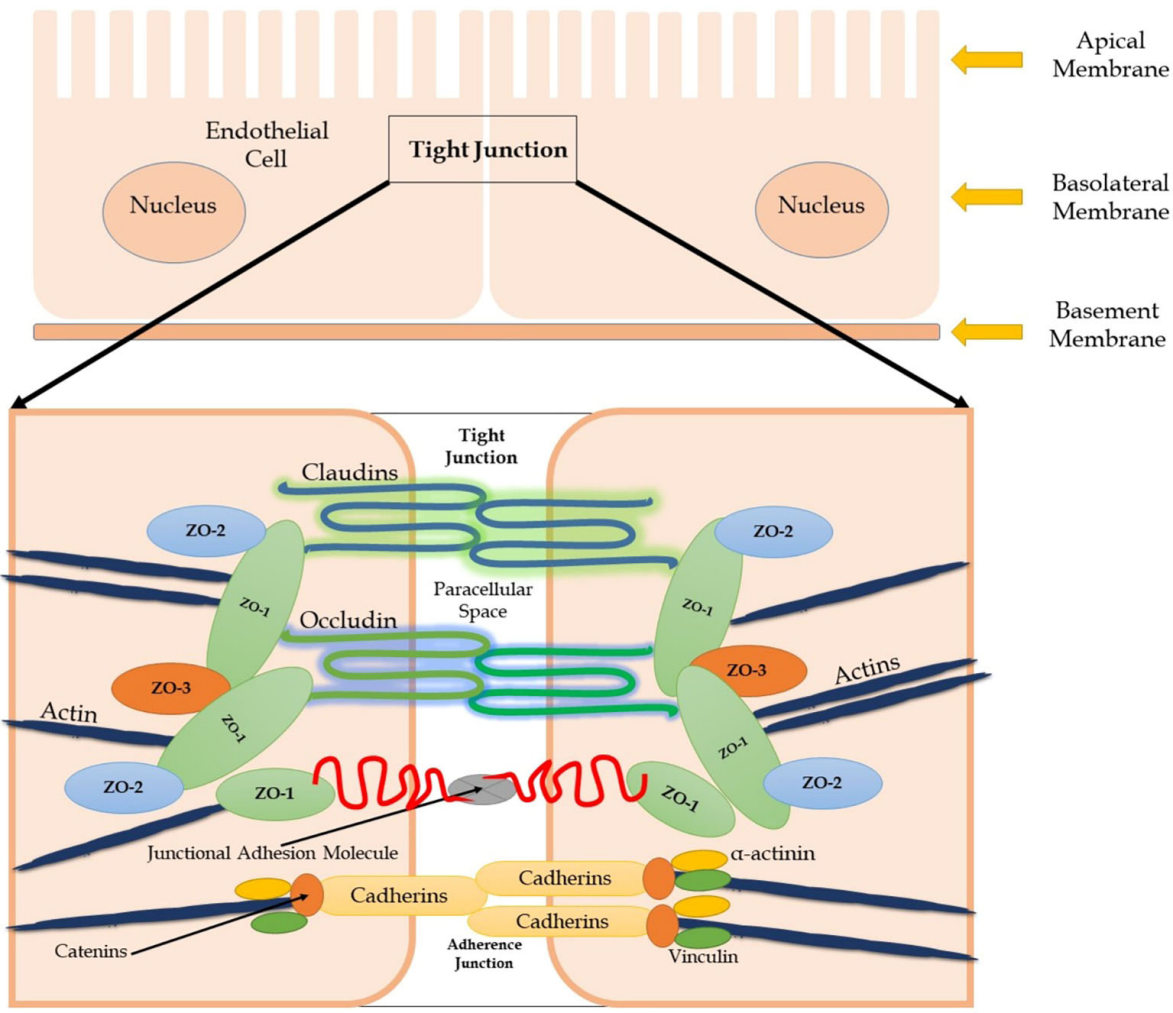
Molecular Structure of Tight Junction Protein Complexes at the Blood-Brain Barrier (The tight junction is formed by several transmembrane proteins including claudins, occludin, and junctional adhesion molecule) on adjacent endothelial cells. The C terminal of transmembrane proteins is bound to cytoskeletal actin by zonula occludens-1 (ZO-1).

The blood–brain barrier (BBB), consisting of four primary cellular elements including cerebral endothelial cells, microglial cells, astrocyte end-feet, and pericytes, is a barrier between the brain parenchyma and blood circulation, providing physiological and anatomical protection for the central nervous system (CNS), shielding the brain from toxic substances in the blood, filtering harmful compounds from the brain back to the bloodstream, and supplying brain tissue with nutrients (1). The cerebral endothelial cells of the brain capillaries regulate vascular permeability, and transport across the brain endothelium is restricted through a defense line in which inter-endothelial tight junctions (TJs) play a crucial role (2) (**Figure 1**). Claudins are integral membrane proteins of the TJs that regulate the function of the TJs. Although claudin-5 is highly expressed in brain endothelial cells, several other tissues also express significant albeit lower levels (e.g., lung, liver, kidney, and skin) (3). In conditions involving increased neuroinflammatory processes, loss of TJ proteins and disruptions in the integrity of the BBB may occur. Loss of BBB integrity facilitates the entry of inflammatory mediators into the CNS (4). In response to increased neuro-inflammation, brain endothelial cells become activated and are characterized by the upregulated expression of cell adhesion molecules to facilitate leukocyte entry to the CNS and facilitate an immune response (5). TJ proteins, especially claudin-5, play an important role in the trans-endothelial migration of peripheral leukocytes, and various upstream signaling components can regulate claudin-5 levels (3, 6). However, anti-inflammatory treatment with glucocorticoids was accompanied by decreased leukocyte expression of claudin-5 (7). Claudin-5 has been studied in psychiatric disorders such as schizophrenia, bipolar disorder, obsessive–compulsive disorder (OCD), attention-deficit/hyperactivity disorder, and autism spectrum disorder, with inconsistencies across studies (8). However, claudin-5 has not yet been investigated in anxiety disorders.

Panic disorder (PD) is a disorder within the anxiety disorder spectrum characterized by recurrent and unexpected panic attacks accompanied by persistent worry and/or avoidance behaviors for 1 month or more (9). It has been reported that the inflammatory response is increased in PD (10), and C-reactive protein (CRP) (11), interleukin-6 (12), interleukin-2 (13), and tumor necrosis factor α levels (12) are higher in subjects with PD compared to healthy control (HC) subjects. Complete blood count (CBC)-derived parameters associated with increased inflammation, such as neutrophil count, white blood cell, and neutrophil-to-lymphocyte ratio, were also found to be higher in subjects with PD compared to HCs (14).

In PD, where an increased inflammatory response is observed, BBB integrity may be affected, and alterations in TJ membrane proteins, which play a key role in BBB integrity, may occur (2, 10). Of note, a review of the literature revealed that claudin-5, a BBB-specific TJ protein found in the BBB endothelium at 600-fold higher concentrations than other claudins (15), has not been studied in PD. This study aimed to compare claudin-5 levels in drug-naïve PD subjects at presentation and after a 6-week follow-up with those in HCs. Our hypothesis was that claudin-5 level is associated with PD symptomatology and that its levels will change as PD symptom severity decreases.

## Materials and methods

### Study design, procedure, and sampling

The PD group of this prospective cohort study consisted of consecutive outpatients diagnosed with PD who were admitted to Health Sciences University Fethi Sekin City Hospital psychiatry outpatient clinic between March 21, 2025, and July 15, 2025. PD diagnosis was made according to the *Diagnostic and Statistical Manual of Mental Disorders*, Fifth Edition, Text Revision (DSM-5-TR) (9).

All subjects admitted to the outpatient clinic with a diagnosis of PD underwent routine biochemical analysis before starting treatment. Based on the biochemical analysis data of the included subjects with PD, psychopharmacological therapy was administered based on the diagnosis of PD, and the PD subjects were recalled for a follow-up visit 6 weeks later. At the initial admission and after 6 weeks of follow-up, blood samples were drawn to measure claudin-5 levels, and the DSM-5 Panic Disorder Scale (PDS) was administered. Sociodemographic and clinical data were recorded at the initial admission.

CBT techniques, including Socratic questioning, cognitive restructuring, relaxation techniques, exposure, systematic desensitization, psychoeducation, and coping strategies, were administered to all subjects with PD. Additionally, psychoeducation was provided to subjects diagnosed with PD, and all were managed with psychopharmacological agents. Psychiatric interview, the application of the study, and the initiation of medical therapy were determined as 30 ± 5 minutes for each patient.

The HC group was formed by hospital staff who donated blood annually as part of routine health screenings, based on their order of presentation. The HC group did not have any psychiatric or organic disease, did not use drugs, did not have alcohol or substance comorbidities, and did not have neurocognitive impairment. Information about the study was provided, and informed consent was obtained from the HC group. In addition to routine biochemical analysis, blood samples were collected from the HC group, whose sociodemographic data were available, to analyze claudin-5 levels.

Psychiatric diagnosis, treatment, and follow-up processes, and the application of study forms (sociodemographic and clinical data form, and PDS) were carried out by the first author, who had 6 years of psychiatric practice experience (DÖ).

The initial admissions for drug-naïve PD subjects (n = 35) were completed on May 30, 2025. The admissions of the PD subjects included in the study (n = 27) after 6 weeks of follow-up were completed on July 15, 2025. The admissions of the HC group (n = 25) were completed between March 25, 2025, and July 10, 2025.

The disease and medication histories of the participants were checked with their consent from the national patient registry system, *e-Nabız*.

### Inclusion and exclusion criteria

**Figure 2:**
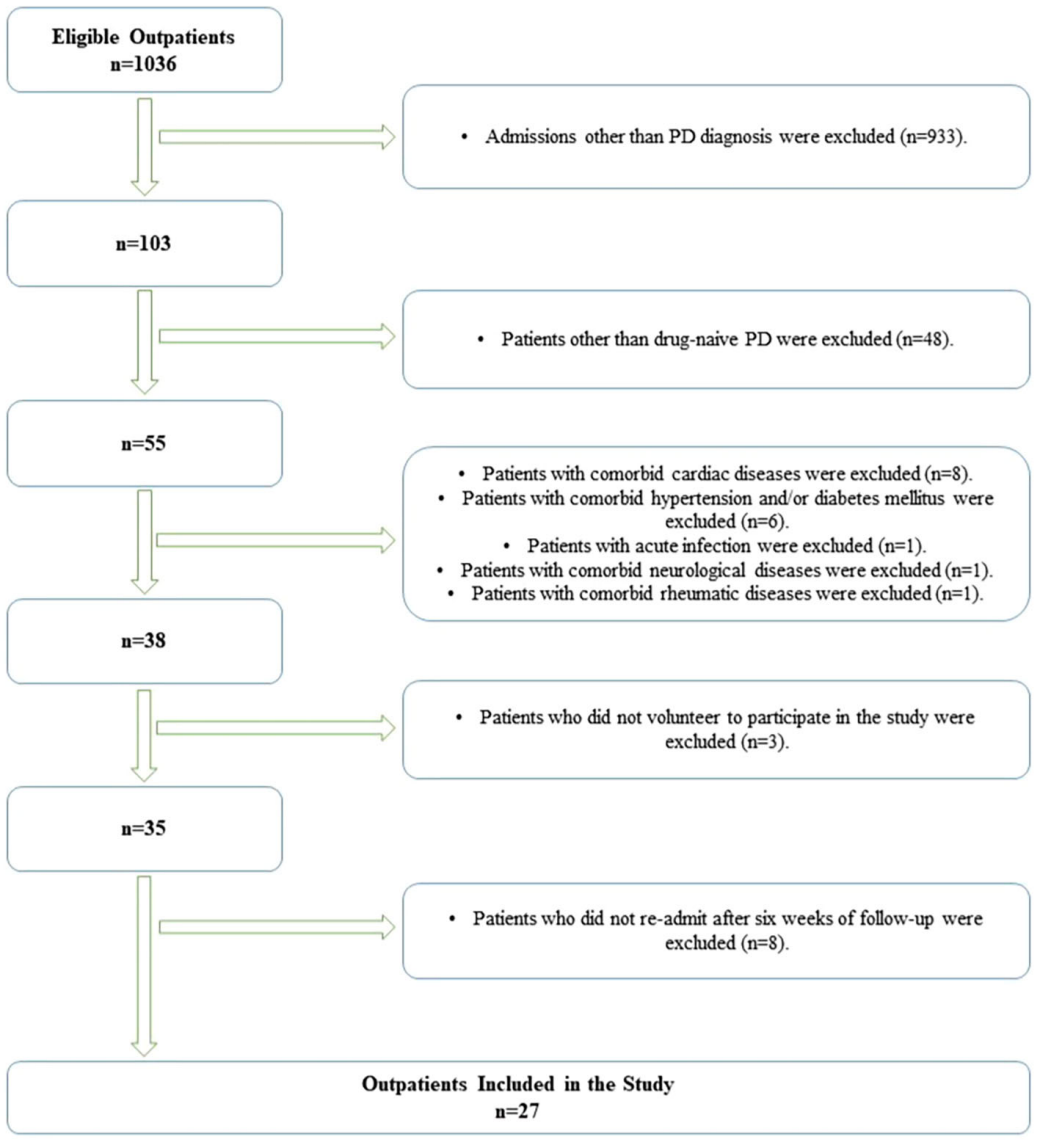
Flowchart of the panic disorder group.

Among all subjects admitted to the psychiatric outpatient clinic, patients with a diagnosis other than PD were excluded from the study. Among subjects with a diagnosis of PD, those with a diagnosis other than drug-naïve PD were excluded. After excluding PD subjects with comorbidities or those who refused to participate in the study, 35 PD subjects were included in the study. Twenty-seven PD subjects were re-examined after a 6-week follow-up, and the necessary procedures for the study were performed. There were no PD and HC subjects with an active alcohol or drug use disorder diagnosis or a history of diagnosis. The process of forming the PD group is illustrated in **Figure 2**.

### Ethical approval and funding

Ethical approval was obtained from the Elazığ Fethi Sekin City Hospital Non-invasive Research Ethics Committee, and the 1964 Declaration of Helsinki was followed (Number 2025/6-4). All respondents consented to the use of their information for research purposes. No funding was declared.

### Instruments

#### Sociodemographic and clinical data form

The sociodemographic data form was developed by the researchers in order to determine the sociodemographic characteristics of the subjects participating in the study. In this form, information about the patient’s name/surname, age, gender, admission date, body mass index (BMI), education level, marital status, working status, residence, smoking status, etc., was queried. Information regarding the PD clinic was obtained from the PD group.

#### DSM-5 panic disorder scale

The PDS is a 10-item self-assessment scale developed by the American Psychiatric Association and provides a 5-point Likert-type rating (0 = never, 1 = sometimes, 2 = half of the time, 3 = most of the time, and 4 = all of the time). When completing the scale, participants considered how much PD has affected their private lives in the last 7 days. The scores obtained from the scale items determined the severity of PD. The score that could be obtained from the scale was between 0 and 40, and high scores indicate that PD is severe. The Turkish validity and reliability study was performed by Balıkçı et al. (16). It assesses the severity of PD in individuals aged 18 and older. The average total score, which is calculated by dividing the total raw score by the number of items in the scale, was used in this study.

A 10-mL venous blood sample was collected from the participants between 10:00 and 12:00 pm, which is the routine visiting hour, by a registered nurse to obtain claudin-5 and routine biochemical data.

### Biochemical analysis

All subjects included in the study were referred to the medical biochemistry laboratory. Ten-milliliter venous blood samples were collected from the participants between 10:00 am and 12:00 pm, which is the routine visiting hour, by a registered nurse to obtain claudin-5 and routine biochemical data. Centrifugation (5,000 rpm for 10 minutes) was performed within 30 minutes, and routine biochemical analysis was performed on the Beckman Coulter AU480 Biochemical Auto-Analyzer (Beckman Coulter, Inc., CA, USA) device located in the biochemistry laboratory of our hospital. Two-milliliter serum was stored at −80°C until analysis of claudin-5.

Human claudin-5 levels were measured via the enzyme-linked immunosorbent assay (ELISA) method using the human claudin-5 ELISA Kit (catalog number 201-12-230; lot number 202505) provided by SunRed Biotechnology (Shanghai, China) in accordance with the manufacturer’s protocol. The manufacturer’s datasheet expresses that the kit uses a sandwich ELISA format with a biotin-labeled detection antibody and pre-coated anti-human claudin-5 antibody. The features of the ELISA kits were as follows: analytical sensitivity, 0.116 ng/mL; assay range, 0.15–30 ng/mL; intra-assay coefficient of variation (CV%), <10; and inter-assay CV%, <12. It was also reported in the specificity section of the product summary that this kit recognizes human claudin-5 in the samples, and no significant interference or cross-reactivity between human claudin-5 and its analogs was observed.

### Statistical analyses

IBM SPSS Statistics for Windows, Version 26.0 (IBM Corp., Armonk, NY, USA) was used for the statistical analysis. Continuous variables and descriptive statistics are presented as mean ± standard deviation/median/mean rank, while categorical variables are presented as frequency. The categorical data were analyzed using the chi-square test. If the expected frequency of one or more cells was less than five and this rate was above 25%, Fisher’s exact test was used instead of the chi-square test. Compliance with normal distribution was determined using the Kolmogorov–Smirnov test. In comparing two groups, the Mann–Whitney U test was used for variables that did not show normal distribution, and the independent samples t-test was used for variables that showed normal distribution. The effects of gender and BMI on the comparison of pre-treatment claudin-5 levels between the PD and HC groups were controlled using ANCOVA (p-value of Levene’s test = 0.331. For confidence interval adjustment, the Bonferroni method was used. Partial correlation analyses were performed by controlling the effects of various numerical variables. Hedges’ *g* (which provides a measure of effect size weighted according to the relative size of each sample) was an alternative when there were different sample sizes. It was calculated for continuous variables and Cramer’s V (more than 2 × 2 contingency)/phi (2 × 2 contingency) for categorical variables. In comparing dependent groups, the paired samples test was used as a parametric test, and the Wilcoxon signed-rank test was used as a non-parametric test. Binary logistic regression analysis was used in PD prediction. While PD diagnosis was considered the dependent variable, pre-treatment claudin-5 level, the aggregate index of systemic inflammation (AISI), and PD history in first-degree family members were considered independent variables in binary logistic regression analysis. In the binary logistic regression analysis, hierarchical regression was applied to control for the effects of age, gender, and BMI on the results. First, the predictive levels of age, gender, and BMI on the dependent variable were determined, and then (using the next tab), the predictive levels of pre-treatment claudin-5 level, the AISI, and PD history in first-degree family members for PD were determined. Receiver operating characteristic (ROC) curve analysis was used to measure the diagnostic test performance of the claudin-5 level before treatment. A p-value less than 0.05 was considered statistically significant.

When calculating the sample size, since there was no previous study with similar characteristics, the data obtained from the pilot administration conducted on 10 participants (five PD and five HC subjects) were used, and it was seen that there should be at least 14 subjects in each group (claudin-5 level of HC = 3.39 ± 2.11; claudin-5 level of PD = 5.64 ± 3.46; alpha = 0.05; beta = 0.20; power = 0.80). After including 27 subjects in the PD group and 25 subjects in the HC group, the obtained data were subjected to power analysis again, and the adequacy of the sample size was confirmed.

## Results

The PD group consisted of 27 subjects (17 women and 10 men), and the HC group consisted of 25 subjects (15 women and 10 men). There was no statistically significant difference between the groups in terms of age (p = 0.763), gender (p = 0.826), education level (p = 0.124), marital status (p = 0.603), lifestyle (p = 0.603), working status (p = 0.483), residence (p = 0.628), smoking status (p = 0.892), and BMI (p = 0.314). PD history in first-degree family members was significantly higher in the PD group (p = 0.001). The sociodemographic characteristics of the subjects and the data regarding the non-psychiatric physicians that the PD group consulted before psychiatry are shown in **Table 1**. Pre-treatment claudin-5 levels were significantly different between the PD and HC groups (p = 0.021). This significance persisted after controlling for the effects of gender and BMI as covariates (p = 0.025).

**Table 1:** Sociodemographic and clinical characteristics of PD and HC groups.

Variables	PD (n = 27) Mean ± SD and median (mean rank) and [n]	HC (n = 25) Mean ± SD and median (mean rank) and [n]	P-value (Kolmogorov-Smirnov p-value)	Hedges’ *g* and Cramer’s V/phi
Age (years)	48.88 ± 10.10	49.68 ± 8.54	0.763^a^ (0.071)	0.51^d^
Gender (female/male)	17/10	15/10	0.826^f^	0.826^e^
Education level (years)	8.00 (29.41)	5.00 (23.36)	0.124^b^ (<0.001)	0.38^d^
Marital status (married/single/widow/divorced)	23/3/0/1	21/2/1/1	0.603^c^	0.906^e^
Living with (partner–husband–wife–child/parent–sibling/alone)	23/2/2	21/1/3	0.603^c^	0.906^e^
Working status (regular/irregular/no working)	12/0/15	10/2/13	0.483^c^	0.746^e^
Residence (rural/urban)	3/24	3/22	0.628^c^	0.920^e^
Smoking (yes/no)	5/22	5/20	0.892^f^	0.892^e^
PD history in first-degree family members (yes/no)	20/7	7/18	0.001^*f^	0.001^e^
BMI (kg/m^2^)	26.19 ± 3.30	26.98 ± 2.16	0.314^a^ (0.200)	0.28^d^
Variables	PD (n = 27)			
Emergency department admission before psychiatry (yes/no)	16/11			
Cardiology outpatient admission before psychiatry (yes/no)	18/9			
Internal medicine outpatient admission before psychiatry (yes/no)	16/11			
Neurology outpatient admission before psychiatry (yes/no)	5/22			
Pulmonology outpatient admission before psychiatry (yes/no)	4/23			
General surgery outpatient admission before psychiatry (yes/no)	2/25			
Family medicine outpatient admission before psychiatry (yes/no)	11/16			
Who referred to psychiatry? (healthcare professionals/patient himself-herself/relative-friend)	12/9/6			
Antidepressant medication (paroxetine/sertraline/escitalopram)	20/5/2			
Benzodiazepine medication (alprazolam/medazepam)	25/2			

Statistical analysis was performed using ^a^independent samples t-test, ^b^Mann–Whitney U test, ^c^Fisher’s exact test, ^d^Hedges’ *g*, ^e^Cramer’s V/phi, and ^f^chi-square analysis.

PD, panic disorder group; HC, healthy control group; SD, standard deviation; BMI, body mass index.

^*^p < 0.05.

**Table 2** presents the comparison of pre-treatment laboratory findings of the PD group with the HC group. Significant differences were found between the pre-treatment PD group and the HC group in terms of various biochemical markers, especially claudin-5 (p = 0.021), neutrophil count (p < 0.001), and CRP (p < 0.001).

**Table 2:** Comparison of serum claudin-5 and routine biochemical markers of the PD group before treatment and the HC group.

Variables	PD (n = 27) Mean ± SD and median (mean rank)	HC (n = 25) Mean ± SD and median (mean rank)	P-value (Kolmogorov-Smirnov p-value)	Hedges’ *g*
Claudin-5 (ng/mL)	4.10 (31.17)	3.30 (21.46)	0.021*^b^ (<0.001)	0.71
Albumin (g/L)	44.29 ± 4.65	44.68 ± 4.21	0.757^a^ (0.099)	0.08
CRP (mg/L)	3.07 (35.33)	1.02 (16.96)	<0.001**^b^ (<0.001)	1.07
WBC (10^3^/μL)	8.25 ± 1.36	7.04 ± 1.25	0.002*^a^ (0.200)	0.92
PLT (10^3^/μL)	236.00 (28.04)	234.00 (24.84)	0.447^b^ (0.001)	0.41
MPV (fL)	8.99 ± 1.03	8.62 ± 1.01	0.191^a^ (0.200)	0.36
LYM (10^3^/μL)	1.41 ± 0.43	1.26 ± 0.30	0.163^a^ (0.200)	0.40
MONO (10^3^/μL)	0.55 ± 0.14	0.44 ± 0.11	0.004*^a^ (0.200)	0.86
NEU (10^6^/μL)	4.84 ± 1.10	3.57 ± 1.14	<0.001**^a^ (0.200)	1.13
PLR	196.53 ± 61.19	193.25 ± 58.97	0.845^a^ (0.200)	0.05
NLR	3.72 ± 1.42	2.93 ± 1.00	0.026*^a^ (0.062)	0.63
NLPR	0.0147 (28.26)	0.0132 (24.60)	0.384^b^ (<0.001)	0.25
NPR	0.0208 (31.70)	0.0156 (20.88)	0.010*^b^ (<0.001)	0.25
CRP/albumin ratio	0.06 (35.15)	0.02 (17.16)	<0.001**^b^ (0.001)	1.08
CRP/LYM ratio	2.13 (35.22)	0.82 (17.08)	<0.001**^b^ (<0.001)	1.09
SII	826.78 (31.41)	676.84 (21.20)	0.015*^b^ (0.005)	0.72
AISI	512.81 ± 228.43	308.57 ± 150.61	<0.001**^a^ (0.093)	1.04
SIRI	2.04 ± 0.86	1.30 ± 0.55	0.001*^a^ (0.200)	0.93

Statistical analysis was performed using ^a^independent samples t-test and ^b^Mann–Whitney U test.

PD, panic disorder group; HC, healthy control group; SD, standard deviation; CRP, C-reactive protein; WBC, white blood cell; PLT, platelet count; MPV, mean platelet volume; LYM, lymphocyte count; MONO, monocyte count; NEU, neutrophil count; PLR, platelet-to-lymphocyte ratio; NLR, neutrophil-to-lymphocyte ratio; NLPR, neutrophil-to-lymphocyte and platelet ratio; NPR, neutrophil-to-platelet ratio; SII, systemic immune inflammation index [(neutrophils × platelets)/(lymphocytes)]; AISI, aggregate index of systemic inflammation [(neutrophils × monocytes × platelets)/(lymphocytes)]; SIRI, systemic inflammation response index [(neutrophils × monocytes)/(lymphocytes)].

*p < 0.05; **p < 0.001.

A significant difference was found between the pre- and post-treatment claudin-5 levels and PDS scores of the PD group (**Table 3**).

**Table 3:** Comparison of serum claudin-5 and PD symptom severity of the PD group before and after treatment.

Variables	PD before treatment (n=27) Mean±SD	PD after treatment (n=27) Mean±SD	P-value
Claudin-5 (ng/mL)	5.45 ± 3.38	3.10 ± 2.19	0.001*^a^
PDS	36.25 ± 3.48	12.92 ± 4.41	<0.001**^b^

Statistical analysis was performed using ^a^Wilcoxon signed-rank test (Z = −3.209) (negative mean rank = 14.66, N = 22; positive mean rank = 11.10, N = 5) and ^b^paired samples test.

PD, panic disorder group; SD, standard deviation; PDS, DSM-5 Panic Disorder Scale.

^*^p < 0.05; ^**^p < 0.001.

The claudin-5 levels of the post-treatment PD group (mean ± SD = 3.10 ± 2.19, mean rank = 24.07, median = 2.80) and the HC group (mean ± SD = 3.46 ± 1.96, mean rank = 24.07, median = 3.30) were compared, and no statistical significance was found (Kolmogorov–Smirnov test p = 0.007, Mann–Whitney U test p = 0.230).

In the PD group, partial correlation analysis was performed by controlling for the effects of age, gender, BMI, smoking status, and antidepressant and benzodiazepine status; a significant relationship was found between the pre-treatment PDS score and pre-treatment claudin-5 level (r = 0.474, p = 0.030), and between the pre-treatment claudin-5 level and pre-treatment CRP (r = 0.661, p = 0.001).

Binary logistic regression analysis was applied to reveal the relationship between the parameters found to be significant and the groups. The significant variables were examined one by one using binary logistic regression analysis. After various modeling attempts, a model was created using the pre-treatment claudin-5 level and the AISI. A hierarchical model was used to control for the effects of age, gender, and BMI on the predictive value of pre-treatment claudin-5 level and the AISI for PD diagnosis. Initially, the presence of a PD diagnosis was added as a dependent variable; age, gender, and BMI were added to the first analysis as independent variables. Then, using the next tab, pre-treatment claudin-5 level and the AISI were added to the second-level analysis as independent variables. According to the binary logistic regression analysis, the sensitivity of these parameters related to determining the participants involved in the PD and HC groups was 70.4%, and the specificity was 76.0% (Beginning block, −2 log-likelihood = 72.010, overall p = 0.757; Block 1, −2 log-likelihood = 70.812, Cox and Snell R^2^ = 0.023, Nagelkerke R^2^ = 0.030, Hosmer and Lemeshow test p = 0.361, Constant p = 0.298; Block 2, −2 log-likelihood = 49.564^a^, Cox and Snell R^2^ = 0.351, Nagelkerke R^2^ = 0.468, Hosmer and Lemeshow test p = 0.203, Constant p = 0.749). The pre-treatment claudin-5 level (p = 0.022) and the AISI (p = 0.003) contributed significantly to the model.

**Figure 3:**
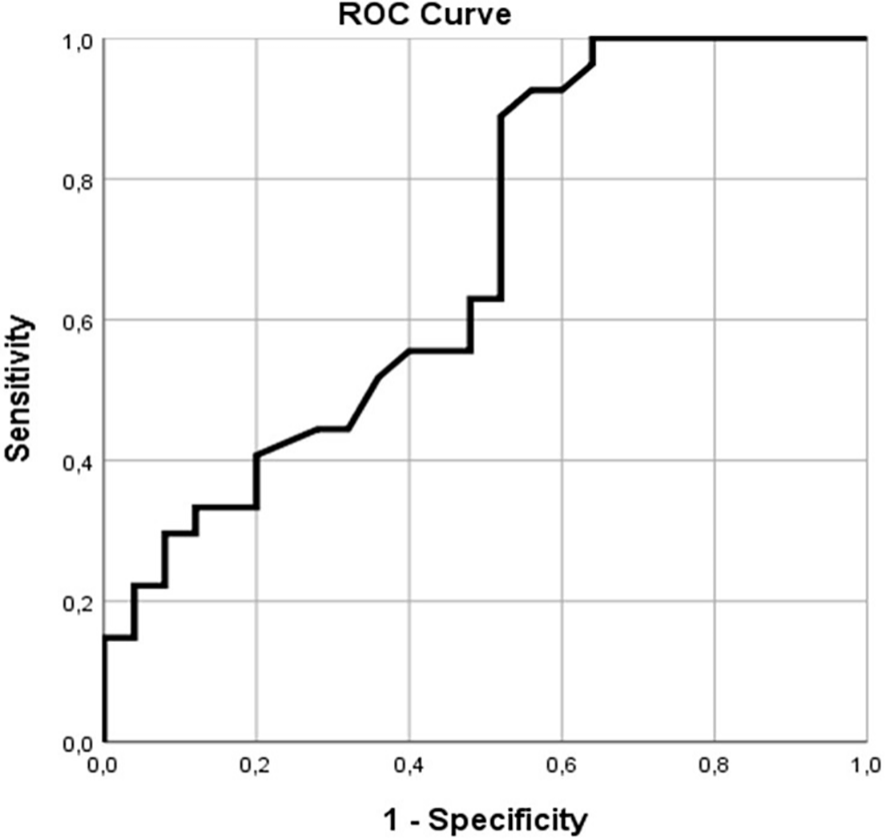
Receiver operating characteristic curve analysis of claudin-5 level before treatment.

ROC analysis of 52 subjects (27 PD and 25 HC) was performed. The area under the ROC curve of claudin-5 level before treatment for PD was 0.687 (p = 0.021, 95% CI = [0.541–0.833]). The optimal cut-off score for claudin-5 level before treatment was 7.85, and its sensitivity and specificity for the diagnosis of PD were 22.2% and 96.0%, respectively (**Figure 3**).

## Discussion

In this study, CRP- and CBC-derived parameters, as well as claudin-5 levels, were examined in drug-naïve PD subjects when they were not under any therapy, and similar examinations were repeated after the subjects were managed with psychopharmacological agents for 6 weeks. Laboratory findings and psychometric scale results were compared before and after treatment and with those of the HC group. Accordingly, claudin-5 levels were higher in PD subjects before treatment than in both the PD subjects after treatment and the HC subjects. In addition to a decrease in PD symptom severity in PD subjects treated with psychopharmacological agents, claudin-5 levels also decreased.

An examination of sociodemographic data reveals that the PD and HC groups were similar in terms of variables that could influence the results, such as age, gender, smoking, and BMI. A history of PD in first-degree relatives was found to be higher in the PD group, and this finding is consistent with the literature (17). Because PD causes physical/somatic symptoms such as palpitations, sweating, numbness, headache, and nausea, referral to non-psychiatric physicians was common in subjects with PD symptoms, especially before psychiatric diagnosis. PD subjects with such physical symptoms most frequently present to emergency departments and cardiology, internal medicine, neurology, and pulmonology units (18). In this study, results compatible with this information in the literature were obtained. Before psychiatric admission, 18 (66.66%) of the PD subjects were admitted to the cardiology department, 16 (59.25%) to the emergency department, and 16 (59.25%) to the internal medicine department. In relation to the high number of subjects consulting non-psychiatric physicians before psychiatric admission, the rate of referral by health professionals among patients admitted to psychiatry was also quite high (44.44%). PD symptom severity was reported to be high in PD subjects before treatment and decreased with treatment. Cognitive behavioral techniques were utilized in addition to psychopharmacological agents in treatment, and PD subjects were informed about the definition, etiology, symptomatology, treatment, and follow-up of PD.

When the study’s findings regarding claudin-5 levels were examined, claudin-5 levels were found to be higher in the pre-treatment PD group compared to the post-treatment PD and HC groups. Claudin-5 levels have been examined in many psychiatric disorders other than anxiety disorders (8). Although OCD is examined under a separate heading in the DSM-5-TR (9), it is a disorder that can be accompanied by anxiety symptoms and is classified as an anxiety disorder in older DSM versions (19). In a cross-sectional study conducted by Kılıç et al. (20) on adults diagnosed with OCD and using psychopharmacological medication, claudin-5 levels were found to be significantly higher than in the HC group. In another cross-sectional study conducted by Işık et al. (21) in children diagnosed with OCD who were using psychotropic medication, claudin-5 levels were found to be significantly higher than in the HC group. In the study conducted by Akın-Kınay et al. (22) on medication-free children diagnosed with OCD, claudin-5 levels were found to be significantly higher than in the HC group. All of these studies (20–22) emphasized that OCD is a disorder characterized by increased neuroinflammation and that increased neuroinflammation may lead to increased BBB permeability. It was suggested that claudin-5 levels, which are abundant in the BBB endothelium and play a key role in BBB permeability (15), may be affected in OCD. To our best knowledge, this is the first study to investigate claudin-5 levels in PD. As in OCD studies where anxiety symptoms can be detected, this study suggested that increased neuroinflammation in PD may increase BBB permeability and that claudin-5 levels may increase in a compensatory manner secondary to the increase in BBB permeability (22). The purpose of this compensatory claudin-5 increase may be to balance BBB permeability and prevent the brain from being affected by possible harmful factors in the general blood circulation (23). Loss of BBB integrity is a prominent and early pathological characteristic of inflammatory conditions and can lead to disruption of fluid homeostasis and facilitate the entry of antibodies, inflammatory mediators, serum and plasma proteins, and anaphylatoxins into the CNS, causing neuroinflammation. BBB permeability increases directly in response to many proinflammatory stimuli, such as tumor necrosis factor α, interleukin-1β, interleukin-6, and lipopolysaccharides, with concomitant downregulation of TJ proteins (3). In the present study, it was deemed more appropriate to explain the findings related to the increase in claudin-5 together with the direct effects of inflammation, in addition to the compensatory/indirect mechanisms in the literature. This conclusion is supported by the study of Dudek et al. (24) in the animal model of stress-induced depressive behavior. Dudek et al. (24) investigated the compensatory responses leading to appropriate behavioral strategies and active resilience and found that stress resilience is linked to both low endothelium expression of the restrictive claudin-5-related transcription factor Forkhead box protein O1 (a transcription factor known to inhibit claudin-5 expression) and permissive epigenetic control of claudin-5 expression. They (24) identified proinflammatory tumor necrosis factor α signaling and histone deacetylase 1 as mediators of stress susceptibility and suggested that pharmacological inhibition of stress-induced increase in histone deacetylase 1 rescued claudin-5 expression in the nucleus accumbens and promoted resilience. They (24) also demonstrated that there were changes in histone deacetylase 1 expression in the nucleus accumbens of subjects not managed with antidepressants, paralleling the loss of claudin-5, and that these deleterious molecular changes were absent in postmortem subjects managed with antidepressants. In this present study, similar to the study by Dudek et al. (24), it was shown that psychopharmacological treatment caused significant changes in claudin-5 levels after 6 weeks.

In this study, unlike other studies (20–22), pre-treatment claudin-5 levels were re-evaluated after a significant decrease in PD symptom severity after 6 weeks of treatment, and it was determined that claudin-5 levels had decreased. No difference was found in claudin-5 levels between the PD and HC groups after treatment. The higher claudin-5 levels found in treated PD groups compared to HC groups in OCD studies may be related to the increased persistence of OCD symptoms despite treatment (22). However, in this present study, the PD group had a lower post-treatment PDS score. The significant correlation between pre-treatment PDS score and claudin-5 levels may also explain how PD symptom severity affects claudin-5 levels through neuroinflammation. Indeed, pre-treatment CRP- and CBC-derived biomarkers such as CRP/albumin ratio, CRP/lymphocyte ratio, neutrophil-to-lymphocyte ratio, neutrophil-to-lymphocyte and platelet ratio, neutrophil-to-platelet ratio, systemic immune inflammation index, the aggregate index of systemic inflammation, and the systemic inflammation response index were found to be significantly higher in the pre-treatment PD group than in the HC group.

### Strengths and limitations

The main strength of this study is its prospective design. Other strengths include the fact that PD subjects were drug-naïve and that their status was examined both medication-free and after 6 weeks of treatment. Another distinguishing feature of this study is that it is the first to examine a psychiatric disorder within the anxiety spectrum.

The most important limitation of this study is the relatively small sample size. The short follow-up period, the lack of re-evaluation of CRP- and CBC-derived parameters associated with neuronal inflammation after 6 weeks of follow-up, and the minor differences in the psychopharmacological agents used are other limitations of this study. Although the sensitivity, assay range, intra-assay CV%, and inter-assay CV% values ​​of the human claudin-5 ELISA kits used in this study were presented, some information (e.g., whether the antibody in the ELISA kit was confirmed using a standard Western blotting test, and whether the antibody was tested against a panel of positive and negative control tissues/cells) could not be obtained through the datasheet. Although the role of pre-treatment claudin-5 level in predicting PD diagnosis was examined using ROC analysis in this study, specificity for PD diagnosis cannot be determined based on ROC analysis alone without including various psychiatric disorders, such as anxiety and mood disorders. Although claudin-5 is the most abundant claudin subtype at the BBB, it is also expressed in tissues outside the BBB, such as the lung and skin. Based on the findings of this study, the proportion of claudin-5 measured in this study associated with the BBB cannot be determined. Furthermore, the effects of potential inflammation and symptomatology in PD on claudin-5 levels in tissues outside the BBB are difficult to compare/distinguish from the effects on claudin-5 levels within the BBB. It is suggested that these limitations be considered in future studies.

## Conclusion

This is the first prospective study to examine claudin-5 in PD, a disorder within the anxiety disorder spectrum, where neuronal inflammation is detected in untreated subjects. Our findings support the hypothesis that increased claudin-5 levels may be the sum of direct and compensatory/indirect responses to impaired BBB permeability in conditions of high PD symptom severity and elevated levels of peripheral biomarkers associated with neuroinflammation. Further studies that increase sample sizes, extend follow-up, and limit the potential effects of medication on outcomes are needed.

## Data Availability

The raw data supporting the conclusions of this article will be made available by the authors, without undue reservation.
